# Effects of inbreeding and genetic modification on *Aedes aegypti *larval competition and adult energy reserves

**DOI:** 10.1186/1756-3305-3-92

**Published:** 2010-10-06

**Authors:** Constantianus JM Koenraadt, Matthias Kormaksson, Laura C Harrington

**Affiliations:** 1 Department of Entomology, Cornell University, Ithaca, NY 14850, USA; 2 Department of Statistical Sciences, Cornell University, Ithaca, NY 14850, USA; 3 Laboratory of Entomology, Wageningen University, P.O. Box 8031, 6700 EH Wageningen, The Netherlands

## Abstract

**Background:**

Genetic modification of mosquitoes offers a promising strategy for the prevention and control of mosquito-borne diseases. For such a strategy to be effective, it is critically important that engineered strains are competitive enough to serve their intended function in population replacement or reduction of wild mosquitoes in nature. Thus far, fitness evaluations of genetically modified strains have not addressed the effects of competition among the aquatic stages and its consequences for adult fitness. We therefore tested the competitive success of combinations of wild, inbred and transgenic (created in the inbred background) immature stages of the dengue vector *Aedes aegypti *in the presence of optimal and sub-optimal larval diets.

**Results:**

The wild strain of *Ae. aegypti *demonstrated greater performance (based on a composite index of survival, development rate and size) than the inbred strain, which in turn demonstrated greater performance than the genetically modified strain. Moreover, increasing competition through lowering the amount of diet available per larva affected fitness disproportionately: transgenic larvae had a reduced index of performance (95-119%) compared to inbred (50-88%) and wild type larvae (38-54%). In terms of teneral energy reserves (glycogen, lipid and sugar), adult wild type mosquitoes had more reserves directly available for flight, dispersal and basic metabolic functions than transgenic and inbred mosquitoes.

**Conclusions:**

Our study provides a detailed assessment of inter- and intra-strain competition across aquatic stages of wild type, inbred, and transgenic mosquitoes and the impact of these conditions on adult energy reserves. Although it is not clear what competitive level is adequate for success of transgenic strains in nature, strong gene drive mechanisms are likely to be necessary in order to overcome competitive disadvantages in the larval stage that carryover to affect adult fitness.

## Background

The incidence of arthropod-borne diseases is increasing globally [1,2]. Control of diseases such as malaria and dengue is complicated by the lack of effective vaccines [3] and new vector control strategies. Genetic modification of arthropods offers a promising strategy for the prevention and control of the diseases they transmit [4-7]. Currently, efforts are underway to develop and evaluate the potential of genetically sterile and disease-refractory *Anopheles gambiae *Giles and *Aedes aegypti *L. mosquitoes [8-10], vectors of malaria and dengue fever, respectively. The goal of this endeavor is to release genetically modified (GM) mosquitoes to either reduce population densities, or replace the wild population with a disease-refractory one [11,12].

Of critical importance is the ability of released GM mosquitoes to survive, mate and pass on desirable genetic traits [13]. GM mosquitoes will have greater success if genetic modification imparts low fitness costs [14]. Fitness is a complex parameter that is impacted by survival and development time of immatures, mating success, adult survival, age of first reproduction and lifetime reproduction [15,16]. Deleterious effects of transgenesis on mosquito fitness may be the result of insertional mutagenesis and/or added burden of the transgene product [14]. Often transgenic insects are inbred to develop a strain that is homozygous for the insertion, further decreasing fitness. Previous studies have not examined the effects of transgene insertion versus the effects of mass-rearing which may lead to the fixation of recessive, fitness-reducing mutations.

An unresolved issue is how competition among the immature stages of wild and GM mosquitoes may affect population reduction or replacement. Irvin et al. [17] reported on larval development rate for transgenic versus a wild type laboratory strain of *Ae. aegypti*, but not at varying (sub-optimal) nutrition levels or with mixtures of transgenic and wild-type mosquitoes, as would eventually occur in nature. Furthermore, other studies have not considered the effects of larval competition and only provided insight into adult survival and fecundity [18,19]. Another drawback of previous studies is that they compared GM strains with highly inbred wild-type strains (the 'outcross' strain).

Therefore, we evaluated the impact of inter- and intra-strain competition on the performance of three strains of *Ae. aegypti*: (1) a wild-type (second generation) strain collected from Mexico (referred to as 'Wild'), (2) Higgs' white eye (HWE), an inbred, white eye mutant strain (referred to as 'Inbred') and (3) a transgenic strain with a green fluorescent protein (GFP) insert (referred to as 'Transgenic'). The latter strain was created using the HWE genetic background. Because breeding sites of *Ae. aegypti*, such as water storage containers and tires, are often food-limited which may lead to density-dependent competition [20-22], we tested how the outcome of competition is altered under optimal and sub-optimal amounts of food. Finally, we investigated differences between wild, inbred and transgenic strains in terms of their energetic reserves upon emergence. These reserves are critical for key behaviors in adult life such as flight, dispersal and mating [23].

## Results

**Figure 1:**
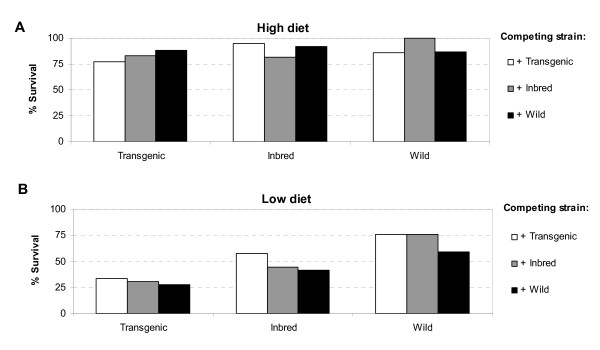
**Survival of *Ae. aegypti *from egg hatch to pupation**. (A) Survival at high diet levels and (B) at low diet levels. Each bar represents one treatment combination consisting of strain and presence of competing strain.

At 'high' diet conditions, survival of larvae to the pupal stage was significantly higher than under 'low' diet conditions (LR-χ^2 ^= 221.47, *df *= 1, *P *< 0.001). At high diet conditions, survival ranged from 78 to 100% and at low diet levels from 27 to 76% (Figure 1). Most strikingly, the survival of Wild larvae in the presence of Transgenic larvae was similar under both diet conditions (86 and 76%, respectively; Figure 1), whereas Transgenic survival in presence of Wild was reduced from 88 to 27%. At high diet levels, a significant interaction was detected, indicating that the strain effect differed over the three levels of competitor presence (Table 1). Post-hoc contrasts of the strain by competitor interaction at high diet level revealed that survival of Inbred larvae was significantly lower in presence of larvae of their own strain compared to survival with any of the two other strains (LR-χ^2 ^= 7.964, *df *= 1, *P *= 0.007). This effect was not significant for the Transgenic and Wild strains (LR-χ^2 ^= 2.725, *df *= 1, *P *> 0.05 and LR-χ^2 ^= 4.231, *df *= 1, *P *> 0.05). At low diet levels, there was no significant interaction, but clear main effects of strain and competitor (Table 1). The effects of both strain and competitor were ranked, with the Wild strain demonstrating higher survivorship than both Inbred and Transgenic (LR-χ^2 ^= 22.963, *df *= 1, *P *< 0.001 and LR-χ^2 ^= 70.813, *df *= 1, *P *< 0.001, respectively), whereas Inbred had significantly higher survivorship than Transgenic (LR-χ^2 ^= 13.248, *df *= 1, *P *< 0.001). A similar ranking was found for the competitor effect whereby Wild had a stronger, negative impact on survival of the other larvae in the well than Transgenic (LR-χ^2 ^= 7.938, *df *= 1, *P *= 0.007). Although the effect of Inbred on survival of the other strains was intermediate, it did not differ significantly from Wild or Transgenic (LR-χ^2 ^= 2.761, *df *= 1, *P *> 0.05 and LR-χ^2 ^= 1.153, *df *= 1, *P *> 0.05, respectively).

**Table 1:** Statistical model results of survival

		Food level			
		High (1.65 mg/larva)		Low (0.825 mg/larva)				
Effect	df	LR-χ^2^	*P*	LR-χ^2^	*P*			
Strain	2	5.506	0.014	71.217	< 0.001
Competitor	2	0.917	0.632	8.069	0.018
Strain * Competitor	4	19.862	0.005	3.278	0.512

LR-χ^2^: Likelihood ratio chi-square value

Effects of strain, presence of competing strain and strain by competitor interaction on survival to the pupal stage of *Ae. aegypti *larvae at 'high' and 'low' diet levels.

**Figure 2:**
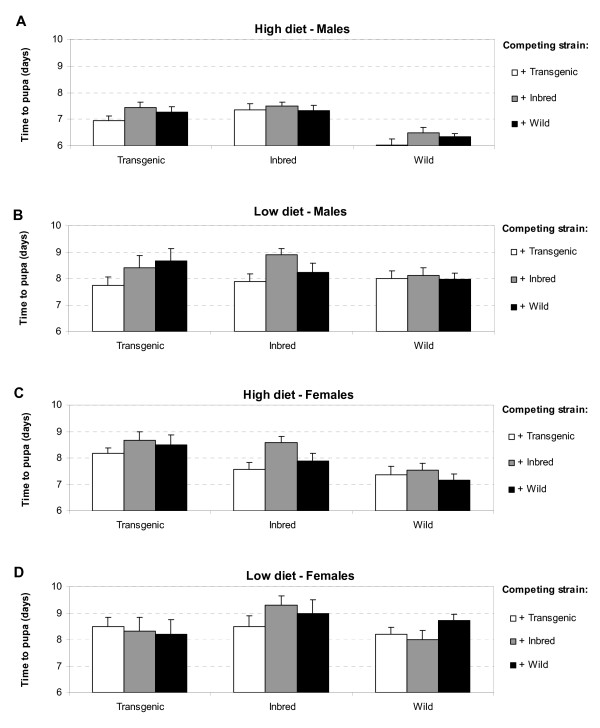
**Least squares means (±SE) of time from egg hatch to pupation (development time) of *Ae. aegypti***. (A) Development time of males at high diet levels, (B) of males at low diet levels, (C) of females at high diet levels and (D) of females at low diet levels. Each bar represents one treatment combination consisting of strain and presence of competing strain.

Because of significant interactions between food or sex and the main variables of interest (strain and competitor), subsequent analyses of development time were conducted separately for the four (2 × 2) different level combinations of food and sex. Wild males at optimal food conditions developed significantly faster to the pupal stage than Inbred or Transgenic males (Tukey HSD, *P *< 0.05; Figure 2-A). This effect disappeared when reared at sub-optimal food conditions (Figure 2-B, Table 2). Under sub-optimal diet conditions all strains of larvae took longer to develop to adults than under optimal diet conditions: Wild larval development time increased by 25-33%, whereas it increased 11-19% for Transgenic and 7-19% for Inbred. A similar trend was observed for females. Under optimal diet conditions, female Wild larvae developed faster into pupae than Inbred or Transgenic larvae (Tukey HSD, *P *< 0.05; Figure 2-C). Moreover, a significant effect of the competing strain was observed. Tukey HSD tests revealed that in the presence of Inbred, females of all three strains developed significantly slower (time to pupation was longer) than in the presence of Transgenic. As with males, effects disappeared at the suboptimal diet levels where no significant differences between strains or effects of competing strain were found (Table 2).

**Table 2:** Statistical model results of development time

		Males				Females						
		High (1.65 mg/larva)		Low (0.825 mg/larva)		High (1.65 mg/larva)							Low (0.825 mg/larva)							
Effect	df	SS	*P*	SS	*P*	SS	*P*	SS	*P*											
Strain	2	68.886	< 0.001	4.189	0.416	50.344	<0.001	7.410	0.094
Competitor	2	6.038	0.084	13.316	0.063	15.670	0.034	0.956	0.733
Strain * Competitor	4	1.431	0.881	9.854	0.389	6.796	0.562	6.580	0.373

Effects of strain, presence of competing strain and their interaction on development time to the pupal stage of male and female *Ae. aegypti *larvae at 'high' and 'low' diet levels.

**Figure 3:**
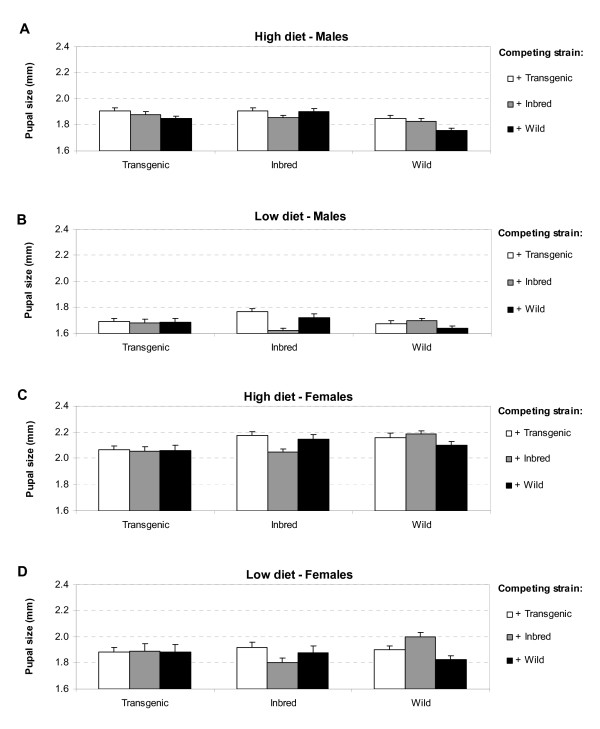
**Least squares means (±SE) of pupal size (cephalothorax length) of *Ae. aegypti***. (A) Pupal size of males at high diet levels, (B) of males at low diet levels, (C) of females at high diet levels and (D) of females at low diet levels. Each bar represents one treatment combination consisting of strain and presence of competing strain.

Because of significant interactions between food or sex and the main variables of interest (strain and competitor), subsequent analyses of pupal size were performed separately for the four different levels of food and sex (similar to the results on development time). For all four models, significant interactions between strain and competitor existed (Table 3), indicating that the effect of competitor presence on pupal size was different for the three strains studied. Subsequent post-hoc analyses revealed that male Wild and Inbred pupae from both diet levels were always smaller when reared in the presence of their own larvae (*t *= 2.01, *P *= 0.046 and *t *= 5.342, *P *< 0.001 at low diet level and *t *= 3.50, *P *< 0.001 and *t *= 2.238, *P *= 0.026 at high diet level, respectively), suggesting higher intra-strain than inter-strain competition. This was also the case for females of the Wild and Inbred strain at high diet levels (*t *= 1.99, *P *= 0.048 and *t *= 3.541, *P *< 0.001) and Wild females at low diet level (*t *= 3.245, *P *= 0.002), but not for Inbred females at low diet level (*t *= 1.932, *P *= 0.0564). Such effects were not observed with Transgenic larvae (Figure 3).

**Table 3:** Statistical model results of pupal size

		Males				Females			
		High (1.65 mg/larva)		Low (0.825 mg/larva)		High (1.65 mg/larva)							Low (0.825 mg/larva)	
Effect	df	SS	*P*	SS	*P*	SS	*P*	SS	*P*					
Strain	2	0.302	< 0.001	0.046	0.119	0.292	0.003	0.032	0.387
Competitor	2	0.121	0.007	0.074	0.033	0.067	0.262	0.024	0.498
Strain * Competitor	4	0.123	0.038	0.281	< 0.001	0.353	0.008	0.224	0.013

Effects of strain, presence of competing strain and their interaction on pupal size of male and female *Ae. aegypti *larvae at 'high' and 'low' diet.

**Figure 4:**
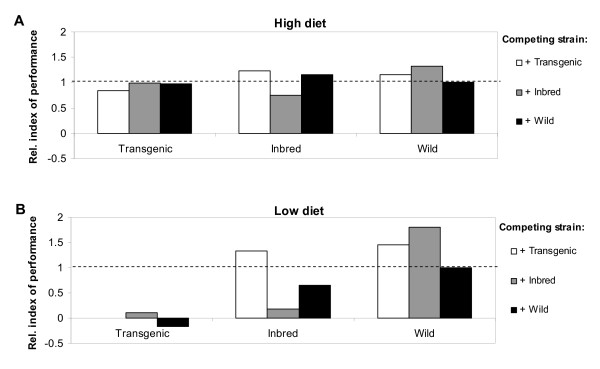
**Relative index of performance for inter- and intra-strain combinations of Transgenic, Inbred and Wild strains**. (A) performance at high diet with the Wild-Wild combination set at 1; (B) performance at low diet with the Wild-Wild combination set at 1.

Figure 4 illustrates the relative index of performance of females after setting the Wild-Wild combination at 1.0 as the reference group. Especially at low diet levels, overall performance of the Transgenic strain was much lower compared to the Inbred and Wild strains. Wild females exposed to the low diet had 38-54% reduction in performance compared to those in high diet treatments. The same comparison demonstrated a 50-88% reduction in Inbred and 95-119% in Transgenic. These results suggest that Transgenic larvae were more sensitive to a change in diet levels than Inbred and Wild, whereas Inbred was more sensitive than Wild.

**Figure 5:**
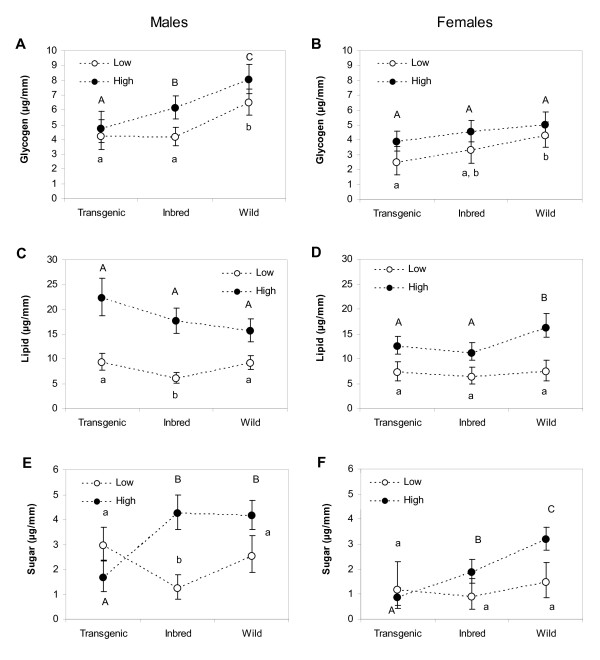
**Average glycogen, lipid and sugar content (μg/mm) of male and female *Ae. aegypti***. Values shown within panels A-F are of the Transgenic, Inbred and Wild strain. Error bars represent 95% confidence limits. Open circles denote the nutritional content at the low diet; closed circles denote nutritional content at the high diet. Averages within a diet level associated with the same letter are not significantly different (LSD post-hoc test, *P *< 0.05).

Glycogen levels of the Wild strain were significantly higher than the Inbred and/or Transgenic strain, except for females at the high diet level (Figures 5-A and 5-B). Inbred males at the low diet level contained significantly less lipids than the Wild and Transgenic strain (Figure 5-C). Wild females reared at high diet levels had significantly more lipids than both the Inbred and Transgenic strain, whereas no significant differences were found at the low diet level (Figure 5-D). At the high diet level, Wild males and females contained significantly more sugars than Inbred and/or Transgenic (Figures 5-E and 5-F). At the low diet level, only male Inbred contained significantly less sugars than Wild and Transgenic (Figure 5-E).

## Discussion

Our study demonstrated lower survivorship and increased development time of the inbred and transgenic strain of *Ae. aegypti *larvae when compared to their wild counterpart. Clearly, this outcome of competition was mediated by the relative competitive strength of each strain as well as the amount of food available. First, the results of increased development time of Inbred and Transgenic larvae were most pronounced at high diet conditions for both males and females. Second, when competition for food was highest (i.e., at the low diet level), the wild-type strain was a superior competitor in terms of survival over the inbred strain, which in turn was superior over the transgenic strain (Figure 1-B). Because nutritional resources in the aquatic environment of *Ae. aegypti *are limited, such effects of competition for nutrients are likely to occur in nature as well [20-22].

Our experimental set-up with microcosms was based on earlier work that investigated the effects of density-dependent competition on the life-history of *Ae. aegypti *[24]. In the field, a substantial diversity in container habitats can be found. These range from large, rain-filled water storage containers (~500 L) to very small containers such as ant-traps filled with tap water. The minimalist approach uses small numbers in small volumes [24], but still with comparable densities as encountered in the field [25]. This experimental design allowed us to track larvae throughout their development and control for the amount of food available per larva.

It is possible that some of the observed effects may offset each other in nature. For example, lower survivorship could result in less frequent interactions with remaining larvae, which in turn could lead to larger, more fecund adults, and eventually result in no net effect on relative fitness. Using the index of performance that incorporates survival, development time and size-related fecundity, we found that the Wild strain had an overall competitive advantage in the larval stage. The Inbred strain was more fit than the Transgenic strain which in turn was less fit than the Wild strain, suggesting that genetic modification imparted a fitness cost on top of inbreeding costs.

The results on energetic reserves of the three strains suggest that inbreeding and genetic modification also affect the mosquito's metabolism. Such differences could be the result of poorer resource acquisition rate during the larval stage (e.g. amount of time spent feeding or efficiency of filter feeding). In general, glycogen reserves for the Wild strain were higher than for the Inbred and/or Transgenic strains. Lipid reserves showed a reverse trend for males, but not significantly so, whereas for females lipids showed a similar trend as glycogen levels. This suggests sex-specific variation in energetic budget as demonstrated for the malaria vector *Anopheles gambiae *[26]. Because glycogen is an important stored energy source for mosquito flight and dispersal that can be rapidly utilized after eclosion, these differences could have a serious impact on mating and eventual reproductive success [23,27].

Although the inbred and transgenic strains had a different origin than the wild strain (Puerto Rico versus Mexico), and intrinsic genetic differences could not be ruled out completely, our results on reduced competitive ability and altered metabolism are most likely the consequence of detrimental effects of both inbreeding and genetic modification. Ideally, transformation should be conducted on genetically diverse laboratory strains which could mitigate the impact of modification and inbreeding [15].

Although it is not clear just how competitive modified mosquitoes need to be compared with wild type mosquitoes in order to replace vector populations, the fitness effects observed in our study are likely to be relevant. First, the strong dependence on food level suggests that the outcome of competition is not fixed and thus fitness evaluations should not be performed only under 'ideal' laboratory conditions [17-19]. Next, our results support earlier statements that incorporation of strong gene drive mechanisms in the transgenic construct will be important [28]. If they are not, a competitive disadvantage in the larval stage may lead eventually to the exclusion of the 'weaker' GM strain in nature [29] ultimately compromising control efforts. For a population reduction strategy based on RIDL or SIT, reducing the potential for density-dependent competition may negate the effectiveness of a mosquito control program, because less competition may result in increased wild type larval survival and potentially more fecund adult mosquitoes. This effect could thereby offset the initial positive effects of a reduced population [30], although the importance of density-dependent competition under field conditions remains unclear [31]. Design of late-acting dominant lethal genetic systems may be promising in this regard as they carry a GM larval population through the density-dependent phase [32].

## Conclusions

Future studies on competition and fitness of transgenic mosquitoes should address how the competitive ability of various transgenic strains is affected when exposed to different scenarios of density-dependent (e.g., container size and food availability) and density-independent factors (e.g., temperature). These studies should address both the fitness cost of inbreeding as well as genetic modification. In line with our results, other studies have shown that expression of genetic background strongly depends on environmental conditions (gene by environment interactions) [33,34]. Similarly, there is an urgent need to evaluate transgenic mosquito lines under more realistic field conditions [13,14] and across life stages.

## Methods

### Mosquito strains

Competition experiments were carried out with combinations of three strains of *Ae. aegypti*: (1) wild-type (Wild); larvae were second generation offspring of field collected pupae from Tapachula, Mexico (14° 54'N, 92° 15'W); (2) Higgs' white eye (HWE), an eye-pigment deficient variant of the Puerto Rican Rexville D strain [35] as the result of a spontaneous mutation (S. Higgs, pers. comm.; [8]) and (3) enhanced green fluorescent protein (EGFP), a HWE strain in which the GFP gene has been inserted through germ-line transformation using the *piggyBac *transposable element (A.A. James, pers. comm.; [36]). All strains were maintained in separate environmental chambers set at a temperature and humidity comparable to the origin of the Wild strain (Tapachula, Mexico): 27°C, 80% RH and a photoperiod of 12:12 L:D. The Inbred (HWE) and Transgenic (GFP) strains were kept at 28°C and 80% RH prior to shipment to our laboratory facilities. Experiments were executed in another environmental chamber with the same temperature, humidity and light settings. Standard rearing conditions for all strains used 200 larvae per tray (27 × 20 × 8 cm) filled with 1 L of tap water. We added 30, 60, 90 and 90 mg of food to the rearing trays on days 0, 1, 3 and 5, respectively (1.65 mg/larva). Food consisted of a 1:1 ratio of lactalbumin: brewers yeast mixture.

### Competition experiments

We followed the approach of Agnew *et al. *[24] to study the effects of larval competition in *Ae. aegypti*. Wells of 12-well cell culture plates (2.2 cm diameter, Corning Incorporated Life Sciences, Lowell, MA) were filled with five ml tap water. Water was added daily to account for evaporation. Newly hatched first-instar larvae (~ 4 h old) were introduced in the following six combinations at high and low diet regimens: (1) 4 Wild larvae, (2) 4 Transgenic larvae, (3) 4 Inbred larvae, (4) 2 Wild plus 2 Transgenic larvae, (5) 2 Wild plus 2 Inbred larvae and (6) 2 Transgenic plus 2 Inbred larvae. We added 0.15, 0.3, 0.6 and 0.6 mg of food per larva on days 0, 1, 3 and 5, respectively, for the high diet regimen (total 1.65 mg/larva). This diet amount was found to be optimal for development of large body size mosquitoes in previous experiments in our laboratory. The low diet consisted of half the amount of the high diet regimen (total 0.825 mg/larva). Mortality was recorded daily and, over the course of the study, the amount of food added to each well was adjusted to the number of larvae remaining. Thirty replicates per treatment were carried out.

Time to pupation was recorded, as well as sex of pupae [37]. Size of pupae was measured as an indicator of adult body size as described by Koenraadt [38]. Briefly, after moving the pupa to its lateral side, cephalothorax length was measured as the distance between the anterior point of the median keel and the ventral tip of the pupal wing sheath [39]. The relationship could be expressed as *y *= 1.110*x *+ 0.014 and *y *= 0.974*x *+ 0.119 for females and males, respectively, whereby *x *= cephalothorax length (mm) and *y *= adult wing length (mm). GFP expression in the eyes of pupae was visualized using a Stemi 2000-C stereomicroscope (Carl Zeiss MicroImaging Inc., Thornwood, NY) equipped with an Endura Bright Royal Blue (450 nm) LED-light (Opto Technology, Inc., Wheeling, IL) and a yellow barrier filter (Edmund Optics Inc., Barrington, NJ). The experiments were terminated when all larvae had died or pupated.

### Nutritional status

To test for differences in nutritional reserves of teneral adults emerging from the single species treatment, we determined glycogen, lipid and sugar content using previously published protocols [40-42]. Body size dependent variation in nutrient quantities were controlled for by expressing nutritional reserves per mm pupal height.

### Data analysis

Our analyses focused on detecting differences in survival to the pupal stage, development time and pupal size that were inherent to the strain (strain effect), how each strain affected survival, development and size of the other larvae in the same wells (competitor effect), and how these effects changed at optimal and sub-optimal diet levels. Random plate and well effects were not found to be significant in our initial model development; consequently, they were removed from further analysis. Survival data were analyzed with the binomial logistic regression procedure (JMP 7.0, Cary, NC, USA). Analyses of development time and pupal size were performed with standard least squares models (JMP 7.0, Cary, NC, USA). Significant main effects in all models without significant interactions were indicative of a 'ranking' in strain and competitor effects. In that case, post-hoc contrasts were specified to test the hypothesis that the Wild strain was 'stronger' than the Inbred strain (e.g., higher survivorship or larger size), that the Inbred strain was stronger than the Transgenic strain and that the Wild strain was stronger than the Transgenic strain. Similarly, we tested the hypothesis that the Wild or Inbred strain *as a competitor *had a greater negative impact on survival, development time or pupal size than the Transgenic strain. Significant interactions between strain and competitor were indicative of intra-strain versus inter-strain differences, i.e. survival, development time and pupal size for larvae reared with their own strain were different than when reared with any of the two other strains. This hypothesis was analyzed by specifying post-hoc contrasts, and significance was evaluated by using Tukey-HSD tests or by correcting significance levels using the Bonferroni correction.

Ideally, the overall impact of transgenesis and inbreeding for cohorts of organisms should be assessed by calculating per capita rates of change. In our study, a simpler index of performance was calculated that combines information about survivorship and fecundity in a way that essentially simulates computation of the per capita rate of change [43]:

$$I=\frac{\ln\frac{1}{N_{0}}\sum_{x}A_{x}{\bar{w}}_{x}}{\sum_{x}xA_{x}{\bar{w}}_{x}/\sum_{x}A_{x}{\bar{w}}_{x}}$$

*N_0 _*represents the initial number of females (assumed to be 50% of the starting number in our experiments); *A_x _*is the number of adult females produced at time *x *of the experiment; ${\bar{w}}_{x}$ represents the size of the emerging female and is a direct proxy for fecundity. For the present study we used female pupal size because of its strong correlation with female adult size [38]. Indices were calculated for each cohort, whereby cohort was defined as all females from one treatment combination. After calculating the indices, we set the value of the Wild-Wild combination at 1 as the reference group. We did this separately for the high diet and the low diet experiment. All other calculated performance index values were then adjusted so that values >1 would indicate better performance and values <1 would indicate poorer performance than the Wild larvae in the presence of larvae of their own strain.

Finally, strain differences in glycogen, lipid and sugar content per unit body size were evaluated for the adults that emerged from the single species experiments. Standard least square regression procedures were used for this purpose. Post-hoc tests were based on least square differences (LSD).

## Competing interests

The authors declare that they have no competing interests.

## Authors' contributions

CJMK carried out the experimental work, analyzed the data and drafted the manuscript. MK participated in statistical data analyses and helped to draft the manuscript. LCH participated in study design and coordination and helped to draft the manuscript. All authors read and approved the final manuscript.

## Funding

This work was funded by a grant from the Foundation for the National Institutes of Health through the Grand Challenges in Global Health Initiative.
